# Correlates of intimate partner violence among urban women in sub-Saharan Africa

**DOI:** 10.1371/journal.pone.0230508

**Published:** 2020-03-25

**Authors:** Chimaraoke O. Izugbara, Mary O. Obiyan, Tizta T. Degfie, Anam Bhatti

**Affiliations:** 1 International Center for Research on Women (ICRW), Washington, DC, United States of America; 2 Department of Demography and Social Statistics, Obafemi Awolowo University, Ife, Nigeria; 3 Department of Reproductive Health & Population Studies, College of Medicine and Health Sciences, Bahir Dar University, Bahir Dar, Ethiopia; 4 Harvard T.H. Chan School of Public Health, Addis Ababa, Ethiopia; University of Cape Coast, GHANA

## Abstract

**Introduction:**

The dynamics of intimate partner violence (IPV)—one of the world’s leading public health problems—in urban Africa remain poorly understood. Yet, urban areas are key to the future of women’s health in Africa.

**Study objectives:**

We explored survivor-, partner-, and household-level correlates of prevalence rates for types of IPV in urban SSA women.

**Method:**

The study uses DHS data from 42,143 urban women aged 15–49 in 27 SSA countries. Associations at the bivariate level were examined using the Pearson Chi-square test. The modified Poisson regression test estimated the relative prevalence of IPV subtypes in the study population at the multivariate level.

**Results:**

Approximately 36% of women in urban SSA experienced at least one form of IPV; 12.8% experienced two types; and 4.6% experienced all three types. SSA urban women who had only primary-level education, had 3 or more living children, were informally employed, were in polygynous unions, or who approved of wife-beating similarly displayed higher adjusted prevalence rates for all three forms of IPV compared respectively to their counterparts without formal education, without a living child, were unemployed, in monogamous unions, or who do not approve of wife-beating. On the other hand, the region’s urban women who began cohabiting between ages 25 and 35 years or who lived in higher wealth households showed consistently lower adjusted prevalence rates for all three forms of IPV relative to their counterparts who began cohabiting before 18 years or who lived in lower wealth households. Compared to their counterparts without formal education, without a living child, or whose partners did not have formal education, women with secondary and higher education, with 1–2 living children, or whose partners had only primary level schooling displayed higher adjusted prevalence rates for both IPEV and IPPV, but not for IPSV. However, relative to their counterparts whose partners were aged 25 years or below, living with a partner aged 40 years and above was associated with statistically significant reduced prevalence rates for IPPV and IPSV, but not for IPEV. Only for IPPV did women with partners educated at secondary and above levels display statistically significant higher adjusted prevalence rates relative to their counterparts with uneducated partners. Also, solely for IPPV did women who began cohabiting between ages 18 and 24 years or whose partners were employed (whether formally or informally) show decreased adjusted prevalence rates relative to their counterparts who started cohabiting before 18 years or whose partners were unemployed. In addition, only for IPSV did women aged 40 years and above or living in middle wealth households show statistically significant reduced adjusted prevalence rates relative to their counterparts aged less than 25 years or living in lower wealth households.

**Discussion and conclusion:**

By 2030, the majority of SSA women will be urban dwellers. Complexities surround IPV in urban SSA, highlighting the unique dynamics of the problem in this setting. While affirming the link between IPV and marital power inequities and dynamics, findings suggest that the specific correlates of prevalence rates for different IPV sub-types in urban SSA women can, at once, be both similar and unique. The contextual drivers of the differences and similarities in the correlates of the prevalence rates of IPV sub-types among the region’s urban women need further interrogation.

## Introduction

Intimate partner violence (IPV) is any behavior, within an intimate relationship, that causes physical, psychological, or sexual harm[1]. It is a leading public health problem and one of the commonest forms of violations experienced by women globally [2]. The implications of IPV are far-reaching, extending beyond women’s physical, emotional, sexual and reproductive health, to encompass their overall well-being, the welfare of their households and communities, and even the economic and social fabric of societies [3]. Among women who suffer intimate partner violence, injuries, visits to health personnel, disabilities and deaths are common [3–5]. Violence corrodes women’s confidence and mental health, hampering their productivity and contribution to development. Abused women often experience emotional distress and tend to consider, attempt, or carry out suicide frequently. They suffer post-traumatic stress syndrome, depression, anxiety, and low self-esteem and other adverse behavioral outcomes such as alcohol and drug abuse, sexual risk-taking, and a higher risk of subsequent victimization [6]. IPV distresses families and communities. It drains household resources, strains family ties, and depresses family members [7,8]. To avoid further violence, abuse and stigma, women survivors of IPV may amend their behaviors to what is acceptable to their aggressors and victimizers, often becoming their own jailers [9].

Although most African countries have assented to many international declarations and developed several national laws that aim to eliminate violence against women, IPV remains widespread in the continent [10,11]. Nearly 40% of ever-partnered women in Africa have experienced physical and/or sexual intimate partner violence at some point in their lives [11]. Urban settings are increasingly critical for efforts to understand and address IPV in Africa. Most of the region is undergoing rapid urban growth under challenging socio-economic conditions. A significant proportion of the future population growth in SSA will occur in urban areas, and by 2050, 50% of the region’s population is expected to be city dwellers [12]. The majority (60%) of residents in SSA’s largest cities—and a swelling proportion of Africans overall—now live in compromised, congested informal settlements, also called slums [13]. The prevalence of IPV against women in SSA is often higher in impoverished urban settlements than in the general urban population[14–16]. Men and boys who live in poor urban communities have also been described as central to the growing epidemic of deadly IPV against women in SSA [17].

With growing realization that the future of global women’s health is urban [18], understanding the dynamics of and tackling IPV in African cities have become both urgent and critical. But while increasing urbanization in the global south may exacerbate women’s exposure to violence and poor health[19–21], the intersections of IPV and urbanization have been ignored or remain poorly studied. Research has associated a variety of socio-economic factors operating at multiple levels (individual, partner, household, and community) with IPV among urban women [15,21–27]. However, these associations are not clear for different IPV subtypes, for different categories of women, and for different countries in the region. Existing IPV studies among urban women in Africa are mainly comparisons of urban and rural areas of Africa [28,29]; focus on the relationship between IPV and health and other outcomes among urban women [7,30], and address poor urban women’s specific risk factors for IPV [31,32]. Few studies explicitly focus the subtypes of IPV among urban women [9]. Even when they do, the bulk of these studies ignore the broader dynamics of IPV within and between countries in SSA and/or use data that is not representative of urban women in the countries and sub-region [9,32,33].

As Africa’s urban population continues to swell, efforts to tackle IPV require robust characterizations of the women at risk for different forms of IPV; grounded analyses of the intersections of factors that expose women to risk; and focused explorations of country- and regional-level dynamics of women’s experiences of IPV forms. The current study is the first major multicounty study of IPV among urban SSA women. It uses comparable, nationally representative data from 27 SSA countries to ask: 1) What are the magnitude and patterns of IPV and its subtypes within and between selected countries in SSA? 2) What factors are associated with the prevalence and experience of IPV subtypes among urban SSA women; and 3) how does the prevalence of IPV subtypes vary within and between countries in SSA? Utilizing a multilevel approach, the study:(a) provides a profile of urban women who experience IPV and its subtypes within and across selected countries in SSA; and (b), tests the central hypothesis that the prevalence of IPV subtypes among urban women differs within and across SSA countries based on survivor-, partner-, and household-level characteristics. The study responds to a growing need for comparative insights on the dynamics of IPV in contexts and spaces that are critical for the future of women’s health, and for evidence to strengthen national, regional and global responses to IPV.

## Method

### Participants and procedures

The study uses pooled data from 42,143 urban women aged 15–49 years in 27 SSA countries who participated in the most recent Demographic and Health Surveys, Version 6 (DHS-6) or Version 7 (DHS-7) in their countries and completed the Domestic Violence Module of the DHS. The DHS is a nationally representative, population-based, cross-sectional survey sponsored by several governmental agencies, including the United States Agency for International Development (USAID) and administered by ICF International. The DHS collects, describes, and publishes evidence on key demographic and health indicators including HIV, nutrition, violence, livelihoods, sexual and reproductive health, and other indicators (The DHS Program, 2017). It relies on a probability sample of households generated from census frames or, in cases where no census frame exists, from a complete list of villages or communities.

Eligibility for the Domestic Violence Module includes being female, 15–49 years of age, able to complete the survey privately, currently or previously married, and/or living with a male partner. All DHS tools are translated and adapted for each country and then piloted in clusters not selected for inclusion in the survey to assess questionnaire quality (ICF International, 2012). Approximately 2,940 (1.5%) of women that were eligible and agreed to participate in the study countries did not complete the interview due to privacy concerns. The 27 countries and timing of the Demographic and Health Surveys used in the current study are shown in Table 1. Patterns of missingness were examined and found to be minimal (0.01% to 2.8%). The response rate among women completing the Domestic Violence Module stood at 98.2%.

**Table 1:** Countries, sample sizes, and timing of the DHSs used in the study.

Countries	Year	Total	Countries	Year	Total
Angola	2015–16	3,609	Mali	2012–13	591
Benin	2017–18	1,503	Mozambique	2011	1387
Burkina Faso	2010	1,927	Namibia	2013	491
Burundi	2016–17	565	Nigeria	2013	7,279
Cameroun	2011	1,576	Rwanda	2014–15	253
Chad	2014–15	215	Senegal	2017	841
Comoros	2012	652	Sierra Leone	2013	1,073
Democratic Republic of Congo (DRC)	2013–14	1,439	South Africa	2016	1,101
Cote d’Ivoire	2011–12	1,704	Tanzania	2015–16	1,836
Ethiopia	2016	632	Togo	2013–14	1,743
Gabon	2012	2,555	Uganda	2016	1,261
Gambia	2013	1,472	Zambia	2013–14	2,871
Kenya	2014	1,296	Zimbabwe	2015	1,577
Malawi	2015–16	694			

### Measures and variables

The outcome variables for this study are experiences of any form of IPV, namely physical violence, sexual violence and emotional violence. The DHS asks several different questions to establish if a woman has suffered a form of IPV–physical, sexual and / or emotional violence. Women who are currently or were formerly married or in union, responded to a set of thirteen questions (described in the outcome variables section of Table 2). Responses to the questions were grouped into either physical, sexual or emotional. Each outcome variable was coded ‘0’ when the respondent did not experience it, and ‘1’, when it was reported as having occurred.

**Table 2:** DHS definition of variables and recodes.

Variable names	DHS Measurement	Analytical codes
**Individual Variables**	Variable measurement	
Age	15–19; 20–24; 25–29; 30–34; 35–39; 40–44; 45–49.	under 25 years; 25–39 years; 40 years +
Education	no education; primary; secondary; higher	none; primary; secondary +
Occupation	not working; professional/technical/managerial; clerical; sales; agricultural employee; household and domestic; services; skilled manual; unskilled manual; others	none; informal employment; formal employment
Age at first cohabitation	single years from age 8 to 49 years	under 18 years; 18–24 years; 25–34 years; 35 years +
No. of living children	single digits from 0 to 14	0 children; 1–2 children; 3–4 children; 5+ children
Approval of wife beating	beating justified if wife goes out without telling husband; beating justified if wife neglects the children; beating justified if wife argues with husband; beating justified if wife refuses to have sex with husband; beating justified if wife burns the food (a positive answer to any of the 5 questions is indicated as a justification for wife -beating).	not supportive of wife-beating; support wife-beating
**Spouse/Partner variables**		
Age	single years of age from 15 years to 93 years	under 25 years; 25–39 years; 40 years +
Education	no education, primary; secondary; higher	‘none; primary; secondary+
Occupation	not working; professional/technical/managerial; clerical; sales; agricultural employee; household and domestic; services; skilled manual; unskilled manual; others	none; informal employment; formal employment
**Household variables**		
Household wealth	poorest; poorer; middle; richer; richest	low; middle; higher
Number of wives	family type: no other wives; other wives from 1 to 9	one; more than one
**Outcome variables**		
Physical violence	spouse ever pushed, shook or threw something at respondent; spouse ever slapped respondent; spouse ever twisted respondent’s arm or pulled her hair; spouse ever punched respondent with fist or something harmful; spouse ever kicked; dragged or beat up respondent; spouse ever tried to strangle or burn respondent; spouse ever threatened respondent with knife/gun or another weapon; spouse ever attacked respondent with knife/gun or another weapon	yes = experience of at least one of the listed acts of violence No = experience of none of the listed acts of violence
Sexual Violence	spouse ever physically forced respondent to have sex when not wanted; spouse ever forced other sexual acts when not wanted by respondent; spouse ever used threats to force sexual acts when not wanted by respondent?	yes = experience of at least one of the listed acts of violence no = experience of none of the listed acts of violence
Emotional Violence	spouse ever said or did something to humiliate respondent in front of others; spouse ever threatened to hurt or harm respondent including close relatives; spouse ever insulted or made respondent feel bad	yes = experience of at least one of the listed acts of violence no = experience of none of the listed acts of violence

Explanatory variables in the study were selected individual-, household- and partner-level socio-economic and demographic factors, including responding women’s age, level of education, employment status, age at first cohabitation, number of children alive and beliefs regarding wife-beating. Other adjusted variables were the age, level of education, and occupation of the responding women’s spouses or partners, the household wealth and union types of the women. Previous studies [8,16,27,34–44] have linked the selected explanatory variables to IPV in a variety of contexts. Operational definitions of all outcome and explanatory variables are presented in Table 2. The ICF International’s institutional review board reviewed and approved the Demographic and Health Surveys (DHS) used in the current study. The surveys were further approved by the national ethics regulatory boards of the different countries in which the studies were implemented. We sought and received formal permission from MEASURE DHS to use the dataset. All DHS datasets are publicly available at https://dhsprogram.com/data/available-datasets.cfm.

## Data analysis

Statistical analyses were run at univariate, bivariate and multivariate levels. Estimates of the prevalence of outcomes and explanatory variables are presented at the univariate level and stratified at the individual, household, partner, and country levels. Associations between and levels of significance of explanatory, adjusted and outcome variables were examined at the bivariate level using Pearson Chi-square tests. A multilevel generalized linear model with Poisson distribution measured associations between individual-, household- and partner-level variables and IPV within and between countries. Following the lead of previous studies on IPV [45–47], we relied on the modified Poisson regression approach to estimate the incidence ratio (IRR) and 95% confidence interval (CI) of experiencing IPV. We adjusted for selected variables at the multivariate analysis level using three models. Model I was restricted to associations between selected individual characteristics and outcome variables at both within- and between-country levels. Model II included partner- characteristics and model I. Model III included Model I, Model II as well as household-level variables to assess effect measure modification. The model was fitted using the Hosmer Lemeshow Test. All analyses incorporated sampling weights to account for complex survey design and the unequal probability of selection for each participant in the survey. Standard errors for cluster sampling of the primary sampling units were also adjusted for using svyset commands in Stata. Before results were interpreted, multicollinearity between explanatory variables was assessed through the variance inflation factors (VIF) at a reference value of 5. All study estimates were weighted appropriately using the weights assigned to the domestic module in the DHS dataset. Significance levels were estimated at p<0.05, p<0.01 and p<0.001. Analysis was conducted using STATA 15.1 (StataCorp, 2015).

## Results

**Fig 1:**
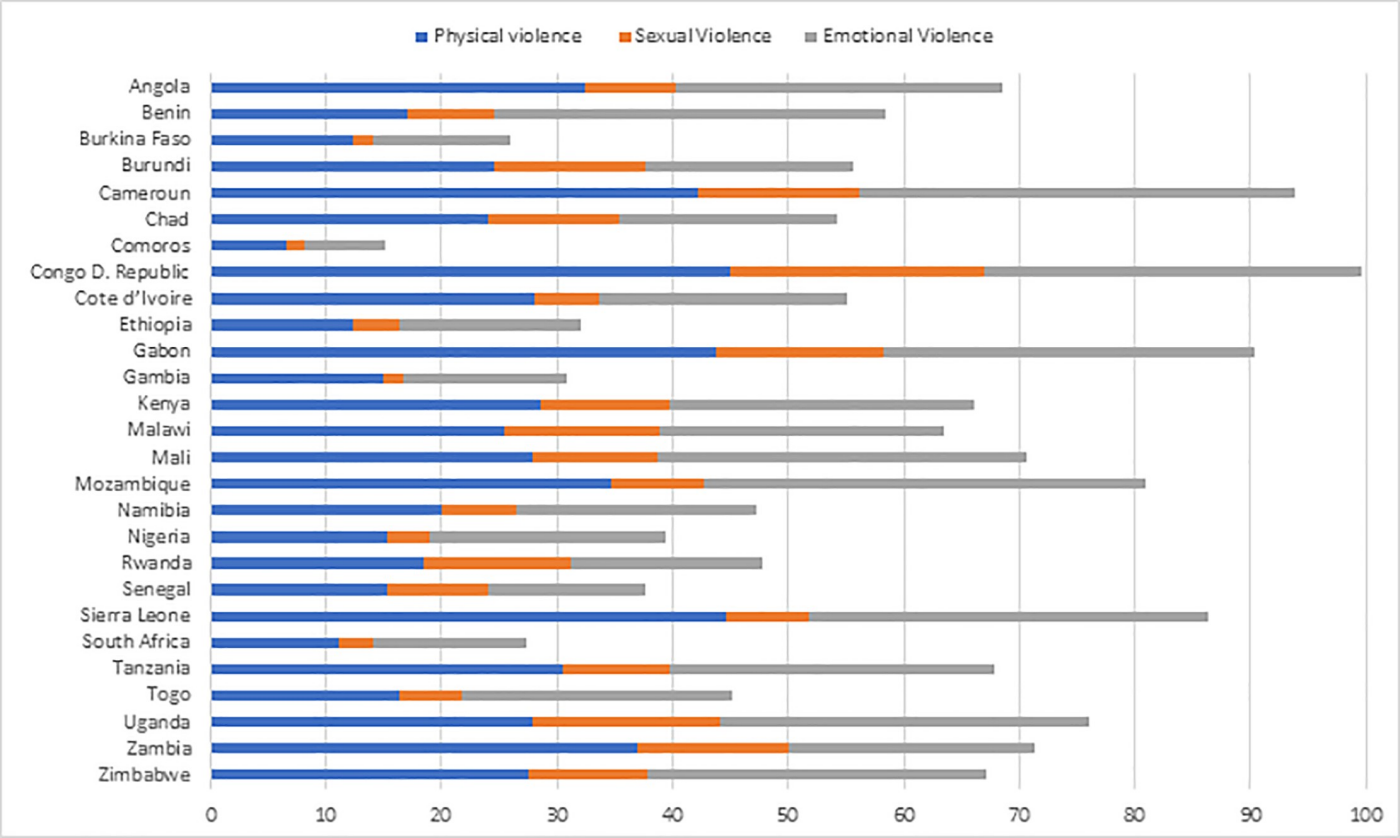
Prevalence of IPV sub-types among urban women in SSA.

Fig 1 shows prevalence estimates of IPV among currently in-union urban women in the study countries. The proportion of urban women who reported at least one form of IPV ranged from 10.8% in Comoros to 56.3% in DRC. Reports of intimate partner physical violence (IPPV) and intimate partner sexual violence (IPSV) were lowest in Comoros (6.5% and 1.7% respectively) and commonest in DRC (45% and 21.9% respectively). The proportion of women reporting intimate partner emotional violence (IPEV) ranged from 7% in Comoros to 38.3% in Mozambique. While the proportion of women who reported all IPV forms was lowest in Comoros (0.4%), it was highest in DRC (11.1%). Overall, 35.7% of women in urban SSA experienced at least one form of IPV; 12.8% experienced two subtypes; and 4.6% of the women experienced all subtypes. (see also S1 Table).

IPPV prevalence rate was lowest among women in the higher wealth category in South Africa (3.4%), and in women aged 40 and above (3.6%) in Comoros. It was highest among women in the lower wealth status in Gabon (55%) and Burundi (51.6%), ranged from 6.9% to 47.9% in women aged 25 to 39 in Comoros and Sierra Leone respectively, and from 3.6% to 44.2% in women aged above 39 years in Comoros and DRC respectively. IPSV was most commonly reported (49.8%) among women in the lowest wealth status in Cote d’ Ivoire and lowest (0.3%) among women aged 40 and above in Comoros; women in the highest wealth category in Gambia (0.4%), and women in formal employment in Ethiopia (0.3%). Prevalence of IPEV was lowest among women in the highest wealth category (3.9%) in Comoros and highest among women in the lowest wealth status in Togo (54.2%) and Cote d’Ivoire (49.8), and among women in informal employment in Cameroun (45.5%) (see S2–S4 Tables).

Bivariate tests of association indicated that while all the explanatory variables of interest were significantly associated with IPPV in Uganda, none was significantly associated with it in Chad and Senegal. In Malawi, Mali, and Sierra Leone, only women’s educational level, women’s occupation, and the number of living children were respectively associated with IPPV. None of the explanatory variables at the bivariate level showed significant association with IPSV in Burkina Faso, Chad, Mali, and Senegal. In Comoros and Sierra Leone respectively, only women’s educational level and women’s approval of wife-beating were significantly associated with IPSV. No association was evident at the bivariate level between the explanatory variables and IPEV in Chad, Mozambique, and Namibia. In Angola, Sierra Leone, and South Africa, only spousal educational level, spousal occupation, and women’s attitude towards wife-beating respectively, were significantly associated with IPEV prevalence. The only variable that was not significantly associated with IPEV prevalence in Burundi was the woman’s age (see S5–S7 Tables).

### Intimate partner physical violence (IPPV)

Regression results for IPPV and selected variables, including IRRs and their associated CIs are provided in Table 3. Women’s educational level was positively associated with an increased prevalence rate for IPPV. Compared to women without formal education, women with primary education had a 27% higher adjusted prevalence rate for IPPV. Women with secondary and higher education also had 1.25 times greater adjusted prevalence rate than that of uneducated women (p <0 .001). Informal employment was associated with significantly heightened rates for IPPV. In Model III, women in informal employment had a prevalence rate for IPPV 1.17 times that of unemployed women (p<0.001). Higher ages (18–34) at first cohabitation were associated with decreased prevalence for IPPV. In the adjusted model, women who began cohabitating between ages 18 and 24 experienced a 5% decreased IPPV prevalence rate relative to women who began cohabiting earlier than their 18^th^ birthday. Also, women who began to cohabit between ages 25 and 34 had a 27% lower adjusted IPPV prevalence rate than those who started cohabiting earlier than 18 years.

**Table 3:** Modified Poisson regression of IPPV on selected characteristics of urban women in sub-Saharan African countries.

Variables	Model I		Model II		Model III	
	IRR	95% C. I.	IRR	95% C. I.	IRR	95% C. I.
**Age—Under 25 years ^ref^**						
**25–39 years**	0.98	0.92–1.05	1.03	0.97–1.10	1.04	0.97–1.11
**40 years +**	0.88	0.80–0.96**	0.97	0.87–1.07	0.98	0.88–1.08
**Level of Education–None ^ref^**						
**Primary**	1.31	1.21–1.41 ***	1.26	1.16–1.36 ***	1.27	1.18–1.38***
**Secondary +**	1.22	1.12–1.32 ***	1.21	1.11–1.31 ***	1.25	1.15–1.36 ***
**Employment Status–None ^ref^**						
**Informal**	1.18	1.12–1.24 ***	1.18	1.11–1.24 ***	1.17	1.11–1.23 ***
**Formal**	0.95	0.85–1.046	0.99	0.89–1.11	1.01	0.90–1.13
**Age of Cohabitation—Under 18 years ^ref^**						
**18–24 years**	0.94	0.90–0.98 **	0.94	0.90–0.99 *	0.95	0.91–1.00*
**25–34 years**	0.71	0.65–0.78 ***	0.72	0.66–0.79 ***	0.73	0.67–0.80 ***
**35 years +**	0.74	0.49–01.13	0.76	0 49–1.17 *	0.76	0.49–1.18
**No. of Children Alive– 0 ^ref^**						
**1–2 children**	1.27	1.16–1.40 ***	1.28	1.16–1.41 ***	1.28	1.16–1.41***
**3–4 children**	1.43	1.29–1.59 ***	1.45	1.30–1.61 ***	1.44	1.30–1.61***
**5 children +**	1.41	1 25–1.58 ***	1.44	1.27–1.62***	1.42	1.26–1.60 ***
**Supports Wife beating–No ^ref^**						
**Yes**	1.32	1.26–1.39 ***	1.31	1.25–1.38***	1.30	1.23–1.36***
**Partner’s Age—Under 25 years ^ref^**						
**25–39 years**			0.93	0.83–1.04	0.93	0.84–1.04
**40 years +**			0.84	0.75–0.95 **	0.84	0.74–0.94**
**Partner’s Level of Education–None ^ref^**						
**Primary**			1.21	1.11–1.30 ***	1.22	1.13–1.32** *
**Secondary +**			1.07	0.99–1.16	1.09	1.01–1.19 *
**Partner’s Employment Status–None ^ref^**						
**Informal**			0.90	0.83–0.98*	0.90	0.83–0.98*
**Formal**			0.81	0.73–0.89***	0.81	0.73–0.90***
**Household Wealth–Lower ref**						
**Middle**					0.95	0.88–1.01
**Higher**					0.88	0.80–0.95**
**No. of wives–one ^ref^**						
**more than one**					1.14	1.08–1.21***
**Country**						
**Angola**	0.84	0.71–0.99 *	0.81	0.69–0.96**	0.79	0.67–0.93**
**Benin**	0.48	0.39–0.59 ***	0.47	0.39–0.58***	0.47	0.38–0.57***
**Burkina Faso**	0.34	0.28–0.42***	0.35	0.28–0.42***	0.36	0.29–0.44***
**Burundi**	0.64	0.53–0.77 ***	0.61	0.50–0.74***	0.64	0.53–0.77***
**Cameroun**	1.06	0.93–1.22	1.05	0.92–1.20	1.04	0.90–1.19
**Chad**	0.60	0.45–0.81 ***	0.62	0.46–0.83**	0.64	0.47–0.86**
**Comoros**	0.20	0.15–0.28 ***	0.20	0.15–0.27***	0.20	0.14–0.27***
**DRC ^ref^**	---	---	---	---	---	---
**Cote D’ivoire**	0.77	0.65–0.92 **	0.79	0.67–0.94**	0.80	0.68–0.95**
**Ethiopia**	0.34	0.24–0.46***	0.33	0.24–0.45***	0.34	0.25–0.47***
**Gabon**	1.16	1.01–1.34*	1.16	1.01–1.33*	1.11	0.96–1.28
**Gambia**	0.42	0.31–0.57***	0.44	0.32–0.60***	0.43	0.32–0.59***
**Kenya**	0.75	0.63–0.89***	0.72	0.61–0.85***	0.72	0.61–0.85***
**Malawi**	0.71	0.53–0.95*	0.69	0.51–0.92*	0.70	0.52–0.94*
**Mali**	0.71	0.58–0.87***	0.66	0.53–0.82***	0.69	0.56–0-.86***
**Mozambique**	0.95	0.81–1.11	0.90	0.77–1.05	0.89	0.77–1.05
**Namibia**	0.62	0.48–0.79***	0.60	0.47–0.77***	0.57	0.44–0.73***
**Nigeria**	0.42	0.35–0.49***	0.42	0.36–0.49***	0.41	0.35–0.49***
**Rwanda**	0.49	0.35–0.68***	0.46	0.33–0.64***	0.48	0.34–0.66***
**Senegal**	0.43	0.33–0.56***	0.44	0.34–0.57***	0.44	0.34–0.57***
**Sierra Leone**	1.15	0.98–1.35	1.18	1.00–1.39*	1.21	1.03–1.42*
**South Africa**	0.38	0.28–0.50***	0.37	0.28–0.48***	0.35	0.27–0.47***
**Tanzania**	0.73	0.62–0.85***	0.68	0.58–0.80***	0.70	0.59–0.82***
**Togo**	0.44	0.37–0.53***	0.43	0.36–0.52***	0.43	0.36–0.52***
**Uganda**	0.69	0.58–0.82***	0.67	0.57–0.79***	0.66	0.56–0.78***
**Zambia**	0.93	0.81–1.08	0.90	0.78–1.04	0.91	0.79–1.05
**Zimbabwe**	0.75	0.64–0.87***	0.73	0.63–0.86***	0.73	0.62–0.86***

*p<0.05

**p<0.01

***p<0.001

Having higher numbers of living children was associated with higher IPPV prevalence rates. In the adjusted model, women with 1–2, 3–4, and 5 + living children had 28%, 44%, and 42% respectively higher prevalence rate of IPPV than women without a living child. Women who approve of wife-beating also had 1.30 times higher adjusted IPPV prevalence rate than those who do not and women whose partners were aged 40 years and above had a 16% lower adjusted prevalence rate for IPPV compared to women whose partners were aged less than 25 years. Interestingly, having a partner with only primary education and having a partner with secondary and higher level of education were both independently associated with an increased IPPV rate in Model III. In the adjusted model, the prevalence rate for IPPV among the former women was 1.22 times (p< 0.001), and among the latter group of women, 1.09 times (p<0.05) greater than women whose partners had no formal education. In the same model, partners’ employment status was also significantly associated with rates for IPPV among women: respondents whose partners were in informal employment and those whose partners were in formal employment respectively had 10% and 19% decreased IPPV prevalence rates compared to women with unemployed partners. While higher household wealth was associated with a decreased adjusted prevalence rate (IRR: 0.88; p:< 0.001) for IPPV, women in polygynous households reported IPPV 14% times more than women in monogamous unions.

In Model I, women in Cameroun, Sierra Leone and Gabon had higher IPPV prevalence rates compared to women in DRC. However, this was only significant in Gabon. In Model II, the reduced IPPV prevalence rates among urban women in the region relative to the DRC counterparts remained generally stable, becoming statistically significant in Sierra Leone and Gabon, but slightly attenuating in significance in Chad. In Model III, the IPPV prevalence rate patterns among urban women in the region relative to their counterparts in DRC were also largely retained, though women in Gabon lost some of their comparative advantages relative to their DRC counterparts.

### Intimate partner sexual violence (IPSV)

Table 4 shows the prevalence rates for IPSV and associated IRRs and CIs. Women aged 40 years and above had a 22% lower adjusted prevalence rate for IPSV compared to their counterparts aged less than 25 years. On the other hand, women with only primary-level education and women in informal employment showed higher adjusted IPSV prevalence rates relative to their uneducated and unemployed counterparts respectively. IPSV prevalence rate was also higher among women with 3 and more living children than in their counterparts with no living child. Compared to those without a living child, women with 3–4 and 5 + living children had 35% and 30% higher prevalence rate respectively for IPSV. Women who approve of wife-beating also had a 63% higher adjusted prevalence rate for IPSV than those who do not. And relative to their counterparts who started cohabiting earlier than their 18th birthday, women who began cohabiting between their 25^th^ and 34^th^ birthdays had a 32% diminished adjusted IPSV prevalence rate.

**Table 4:** Modified Poisson regression of IPSV on selected characteristics of urban women in sub-Saharan African countries.

Variables	Model I		Model II		Model III	
	IRR	95% C. I.	IRR	95% C. I.	IRR	95% C. I.
**Age—Under 25 years ^ref^**						
**25–39 years**	0.89	0.78–1.01	0.94	0.82–1.08	0.96	0.84–1.10
**40 years +**	0.66	0.55–0.80***	0.76	0.61–0.94**	0.78	0.63–0.96*
**Level of Education–None ^ref^**						
**Primary**	1.31	1.13–1.53***	1.28	1.09–1.52**	1.33	1.12–1.57***
**Secondary +**	1.11	0.95–1.30	1.11	0.92–1.33	1.20	0.99–1.45
**Employment Status–None ^ref^**						
**Informal**	1.31	1.18–1.44***	1.31	1.19–1.45***	1.30	1.17–1.43***
**Formal**	0.99	0.80–1.23	1.02	0.82–1.26	1.04	0.84–1.29
**Age of Cohabitation—Under 18 years ^ref^**						
**18–24 years**	0.93	0.84–1.03	0.94	0.85–1.03	0.95	0.86–1.05
**25–34 years**	0.66	0.55–0.80***	0.67	0.56–0.81	0.68	0.57–0.83***
**35 years +**	1.45	0.86–2.46	1.49	0.87–2.54	1.50	0.87–2.57
**No. of Children Alive– 0 ^ref^**						
**1–2 children**	1.15	0.97–1.36	1.16	0.98–1.38	1.16	0.98–1.38
**3–4 children**	1.33	1.10–1.60**	1.36	1.13–1.64***	1.35	1.11–1.63**
**5 children +**	1.27	1.03–1.58*	1.33	1.07–1.66**	1.30	1.05–1.62*
**Supports Wife beating–No ^ref^**						
**Yes**	1.67	1.52–1.85***	1.66	1.50–1.84***	1.63	1.47–1.80***
**Partner’s Age—Under 25 years ^ref^**						
**25–39 years**			0.89	0.74–1.07	0.90	0.99–1.35
**40 years +**			0.76	0.61–0.95*	0.76	0.61–0.95*
**Partner’s Level of Education–None ^ref^**						
**Primary**			1.13	0.96–1.32	1.15	0.99–1.35
**Secondary +**			1.01	0.85–1.19	1.06	0.90–1.25
**Partner’s Employment Status–None ^ref^**						
**Informal**			0.91	0.77–1.08	0.91	0.72–0.94
**Formal**			0.89	0.72–1.10	0.91	0.73–0.84
**Household Wealth–Lower ^ref^**						
**Middle**					0.82	0.72–0.94**
**Higher**					0.71	0.61–0.84***
**No. of wives–one ^ref^**						
**more than one**					1.21	1.08–1.35***
**Country**						
**Angola**	0.46	0.35–0.60***	0.44	0.33–0.58***	0.42	0.32–0.55***
**Benin**	0.46	0.34–0.63***	0.45	0.33–0.61***	0.44	0.32–0.59***
**Burkina Faso**	0.10	0.06–0.17***	0.10	0.06–0.17***	0.11	0.06–0.18***
**Burundi**	0.74	0.55–0.99*	0.70	0.52–0.94**	0.77	0.57–1.04
**Cameroun**	0.75	0.59–0.95*	0.74	0.58–0.94*	0.74	0.58–0.94*
**Chad**	0.57	0.35–0.95*	0.58	0.35–0.97*	0.63	0.37–1.04
**Comoros**	0.12	0.06–0.23***	0.12	0.06–0.23***	0.11	0.06–0.21***
**DRC ^ref^**	---	---	---	---	---	---
**Cote d’Ivoire**	0.31	0.21–0.46***	0.31	0.21–0.46***	0.33	0.22–0.49***
**Ethiopia**	0.24	0.13–0.44***	0.23	0.12–0.42***	0.25	0.14–0.47***
**Gabon**	0.83	0.63–1.08	0.82	0.63–1.07	0.74	0.57–0.97*
**Gambia**	0.10	0.06–0.17***	0.10	0.06–0.18***	0.10	0.06–0.17***
**Kenya**	0.64	0.47–0.86**	0.61	0.45–0.83**	0.61	0.45–0.82***
**Malawi**	0.91	0.65–1.26	0.87	0.63–1.21	0.91	0.66–1.25
**Mali**	0.56	0.39–0.80***	0.53	0.35–0.63***	0.58	0.39–0.87**
**Mozambique**	0.50	0.38–0.67***	0.47	0.35–0.63***	0.47	0.35–0.63***
**Namibia**	0.46	0.30–0.71***	0.44	0.29–0.68***	0.40	0.26–0.62***
**Nigeria**	0.22	0.17–0.30***	0.22	0.17–0.30***	0.22	0.16–0.30***
**Rwanda**	0.76	0.43–1.35	0.71	0.39–1.28	0.76	0.42–1.37
**Senegal**	0.54	0.37–0.79**	0.54	0.37–0.81**	0.55	0.37–0.81**
**Sierra Leone**	0.38	0.25–0.59***	0.39	0.25–0.60***	0.41	0.28–0.63***
**South Africa**	0.27	0.14–0.51***	0.26	0.14–0.49***	0.24	0.13–0.45***
**Tanzania**	0.46	0.35–0.59***	0.43	0.33–0.56***	0.45	0.35–0.59***
**Togo**	0.33	0.25–0.46***	0.33	0.24–0.45***	0.34	0.25–0.46***
**Uganda**	0.87	0.68–1.12	0.84	0.65–1.08	0.82	0.64–1.05
**Zambia**	0.73	0.58–0.92**	0.71	0.56–0.90**	0.72	0.57–0.92**
**Zimbabwe**	0.68	0.53–0.87**	0.67	0.52–0.86**	0.67	0.52–0.86**

*p<0.05

**p<0.01

***p<0.001

The prevalence of IPSV was lower in women whose partners were aged 40 years and above than in women whose partners were aged less than 25 years. The former group women had a 24% lower adjusted prevalence of IPSV compared to the latter. Women in middle and in higher wealth households also had decreased IPSV prevalence rates (IRR: 0.82, p<0.01; and IRR: 0.77, p<0.001) respectively) compared to their counterparts in lower wealth households. However, being in a polygynous union increased IPSV prevalence rate in women by 21% compared to living in monogamous households.

In Model I, urban women in the region generally showed significantly reduced prevalence rates of IPSV compared to their counterparts in DRC, except in Gabon, Malawi, Rwanda, and Uganda. In Model II, while IPSV prevalence rates remained significantly stable across the countries, Burundian women displayed a sturdier reduced IPSV prevalence rate compared to their DRC counterparts. In Model III, with the exceptions of Burkina Faso, Burundi and Chad, the region’s urban women generally retained their statistically significant reduced IPSV prevalence rates compared to their counterparts in DRC.

### Intimate partner emotional violence (IPEV)

Table 5 shows regression results for IPEV prevalence rates and selected variables. Controlling for all variables, women with only primary-level education or with secondary and above levels of education respectively had 25% or 28% higher IPEV prevalence rates than their uneducated counterparts. The higher prevalence rate for IPEV among women in informal employment, compared to their unemployed counterparts, found in Models I (IRR: 1.17, p <0.001) and II (IRR: 1.16, p <0.001) remained significant in Model III (IRR: 1.15, p <0.001). Across the models, women who began cohabiting between their 25th and 34^th^ birthdays retained consistently decreased prevalence rates for IPEV relative to those who began cohabiting earlier than their 18^th^ birthday. In Models I, II and III, the former group of women had 19%, 18% and 18% decreased prevalence rate for IPEV compared to the latter group. Numbers of living child were associated with increased IPEV prevalence rates: women with 1–2, 3–4, and 5 + living children had 27%, 46%, and 43% respectively higher adjusted prevalence rate of IPPV than women without a living child. Also, the higher prevalence rates for IPEV among women who approve of wife-beating, compared to their counterparts who do not, found in Models I (IRR: 1.29, p <0.001) and II (IRR: 1.28, p <0.001) remained statistically significant, diminishing only slightly in the full model (IRR: 1.26, p <0.001). On the other hand, the higher IPEV prevalence rate among women whose partners had only primary-level education relative to women with uneducated partners rose from 19% in Model II to 21% in Model III. Further, while women in higher wealth households showed decreased adjusted IPEV prevalence rates (IRR: 0.86, p<0.001) relative to their counterparts from lower wealth households, being in polygynous unions amplified women’s IPEV prevalence rate by 14% compared to their monogamous counterparts.

**Table 5:** Modified Poisson regression of IPEV on selected characteristics of urban women in sub-Saharan African countries.

Variables	Model I		Model II		Model III	
	IRR	95% C. I.	IRR	95% C. I.	IRR	95% C. I.
**Age-Under 25 years ^ref^**						
**25–39 years**	1.03	0.96–1.10	1.04	0.97–1.12	1.05	0.98–1.12
**40 years+**	0.97	0.88–1.06	0.98	0.89–1.09	0.99	0.90–1.10
**Level of education–None ^ref^**						
**Primary education**	1.27	1.18–1.38***	1.23	1.14–1.34***	1.25	1.16–1.36***
**Secondary +**	1.23	1.13–1.33***	1.23	1.13–1.34***	1.28	1.17–1.40***
**Employment status- None ^ref^**						
**Informal**	1.17	1.10–1.24***	1.16	1.10–1.23***	1.15	1.09–1.22***
**Formal**	1.03	0.92–1.14	1.05	0.94–1.18	1.07	0.95–1.19
**Age of Cohabitation—Under 18 years ^ref^**						
**18–24 years**	0.99	0.94–1.04	0.99	0.95–1.04	1.00	0.95–1.05
**25–34 years**	0.81	0.74–0.89***	0.82	0.75–0.89***	0.82	0.76–0.90***
**35 years +**	0.79	0.53–1.17	0.80	0.53–1.19	0.80	0.53–1.20
**No. of Children Alive– 0 ^ref^**						
**1–2 children**	1.27	1.15–1.40***	1.27	1.15–1.40***	1.27	1.15–1.40***
**3–4 children**	1.47	1.32–1.64***	1.47	1.31–1.64***	1.46	1.31–1.63***
**5 children +**	1.46	1.30–1.65***	1.45	1.28–1.64***	1.43	1.27–1.62***
**Supports Wife beating–No ^ref^**						
**Yes**	1.29	1.22–1.36***	1.28	1.21–1.35***	1.26	1.20–1.33***
**Partner’s Age—Under 25 years ^ref^**						
**25–39 years**			0.96	0.85–1.08	0.96	0.85–1.08
**40 years +**			0.95	0.84–1.09	0.95	0.83–1.08
**Partner’s Level of Education–None ^ref^**						
**Primary**			1.19	1.09–1.29***	1.21	1.11–1.31***
**Secondary +**			1.04	0.96–1.14	1.08	0.99–1.18
**Partner’s Employment Status–None ^ref^**						
**Informal**			0.96	0.87–1.06	0.96	0.87–1.06
**Formal**			0.89	0.80–1.00	0.90	0.81–1.01
**Household Wealth–Lower ^ref^**						
**Middle**					0.94	0.86–1.02
**Higher**					0.86	0.79–0.94***
**No. of wives–one ^ref^**						
**more than one**					1.14	1.07–1.22***
**Country**						
**Angola**	0.98	0.81–1.19	0.97	0.80–1.18	0.94	0.77–1.25
**Benin**	1.28	1.09–1.49***	1.26	1.08–1.48***	1.33	1.06–1.46**
**Burkina Faso**	0.44	0.35–0.57***	0.44	0.35–0.57***	0.46	0.36–0.59***
**Burundi**	0.63	0.51–0.77***	0.60	0.49–0.74***	0.63	0.51–0.78***
**Cameroun**	1.29	1.13–1.49***	1.27	1.10–1.46***	1.25	1.08–1.44**
**Chad**	0.66	0.47–0.94*	0.67	0.47–0.95*	0.69	0.49–0.98*
**Comoros**	0.29	0.22–0.38***	0.28	0.22–0.37***	0.28	0.21–0.36***
**DRC ^ref^**	---	---	---	---	---	---
**Cote d’Ivoire**	0.80	0.65–0.98*	0.80	0.65–0.99*	0.81	0.66–1.00*
**Ethiopia**	0.59	0.44–0.76***	0.56	0.43–0.74***	0.59	0.45–0.78***
**Gabon**	1.13	0.96–1.34	1.13	0.95–1.33	1.07	0.90–1.27
**Gambia**	0.54	0.40–0.71***	0.54	0.41–0.72***	0.54	0.40–0.71***
**Kenya**	0.92	0.78–1.10	0.89	0.75–1.06	0.89	0.75–1.06
**Malawi**	0.93	0.71–1.19	0.90	0.70–1.17	0.92	0.71–1.20
**Mali**	1.14	0.90–1.43	1.10	0.85–1.41	1.15	0.90–1.48
**Mozambique**	1.45	1.24–1.69***	1.38	1.18–1.62***	1.38	1.18–1.61***
**Namibia**	0.82	0.65–1.04	0.80	0.64–1.02	0.76	0.60–0.97*
**Nigeria**	0.74	0.62–0.87***	0.73	0.61–0.87***	0.72	0.60–0.86***
**Rwanda**	0.58	0.43–0.79***	0.55	0.40–0.74***	0.57	0.42–0.78***
**Senegal**	0.52	0.39–0.69***	0.52	0.39–0.69***	0.51	0.38-.69***
**Sierra Leone**	1.21	1.01–1.46*	1.23	1.02–1.48*	1.26	1.05–1.52**
**South Africa**	0.58	0.45–0.74***	0.56	0.44–0.73***	0.54	0.42–0.70***
**Tanzania**	0.92	0.77–1.10	0.86	0.72–1.03	0.89	0.74–1.06
**Togo**	0.85	0.70–1.03	0.83	0.69–1.01	0.84	0.69–1.01
**Uganda**	1.08	0.91–1.249	1.05	0.88–1.25	1.03	0.87–1.23
**Zambia**	0.73	0.62–0.86***	0.71	0.61–0.84***	0.72	0.61–0.85***
**Zimbabwe**	1.06	0.90–1.23	1.04	0.89–1.22	1.04	0.89–1.22

*p<0.05

**p<0.01

***p<0.001

In Model I, only in Benin, Cameroun, Gabon, Mozambique, and Sierra Leone did urban women display statistically significant higher IPEV prevalence rates compared to their DRC counterparts. In the same model, urban women in 12 of the countries (Burkina Faso, Burundi, Chad, Comoros, Cote d’Ivoire, Ethiopia, Gambia, Nigeria, Rwanda, Senegal, South Africa and Zambia) had significantly decreased IPEV prevalence rates compared to their counterparts in DRC. These patterns were largely retained in Models II and III. However, it was only in Model III that Namibian women joined the bulk of the region’s women to experience a statistically significant reduced IPEV prevalence rate (IRR:0.76, p<0.05) relative to their DRC counterparts.

## Discussion

This paper is arguably the first truly regional exploration of prevalence rates for IPV subtypes among urban women across SSA. By focusing on IPV subtypes and their particular correlates in urban women across 27 countries in SSA, the study constitutes a major shift from research that treats IPV as an invariable phenomenon or focuses only on a few of its forms in few African contexts [5,32,33,48]. Some notable patterns and findings emerge from the study. Descriptive findings showed critical patterns in the extent, magnitude and correlates of IPV forms in different countries and among diverse groups of women in SSA. In general, urban IPV rates among urban women in the countries we studied closely mirror prevalence rates of IPV globally, nationally and regionally. Globally, over a third (35%) of women have experienced physical and/or sexual violence by a partner or non-partner. A WHO study showed that the global lifetime prevalence of IPV among ever-partnered women for Africa was nearly 40% [11]. In Nigeria, estimates put the prevalence of IPV at 31% to 61% for psychological/emotional violence, 20% to 31% for sexual violence, and 7% to 31% for physical violence [49]. While IPPV has been the focus of many studies, the current research shows that IPEV and IPSV are also common in urban areas in SSA. The low report for IPSV should, however, be interpreted with caution. IPSV remains a taboo topic in many African contexts. As a result, few survivors feel comfortable to report it in surveys [50].

With regards to IPV in urban SSA, Comorian women were safest while women in DRC were the least unsafe. The proportion of urban women in SSA who report IPV is significantly high in conflict and post-conflict states such as DRC, Uganda, and Sierra Leone. Conflict and post-conflict situations increase women’s risk for violence within and outside unions [47,51–55]. Social protection mechanisms deteriorate at multiple levels during conflicts, intensifying the conditions and circumstances that expose women to risks for violence [40,56].

Our findings indicate the unique dynamics of IPV in urban SSA. To illustrate, only five correlates—having only primary-level education, having 3 or more living children, being informally employed, being in polygynous unions, or supporting wife-beating—were commonly associated with higher adjusted prevalence rates for all three forms of IPV. Conversely, two variables–starting to cohabit between ages 25 and 35 years or living in higher wealth households showed consistently lower adjusted prevalence rates for all three forms of IPV. Relative to their counterparts without formal education, without a living child, or whose partners did not have formal education, women with secondary and higher education, with 1–2 living children, or whose partners had only primary level schooling exhibited higher adjusted prevalence rates for both IPEV and IPPV, but not for IPSV. Also, in comparison with their counterparts whose partners were aged 25 years or below, living with a partner aged 40 years and above was statistically associated with reduced prevalence rates for IPPV and IPSV, but not for IPEV. Only for IPPV did women with partners educated at secondary and above levels display statistically significant higher adjusted prevalence rates relative to their counterparts with uneducated partners. Only for IPPV too did women who began cohabiting between ages 18 and 24 years or whose partners were employed (whether formally or informally) show decreased adjusted prevalence rates relative to their counterparts who started cohabiting before 18 years or whose partners were unemployed. However, it was only for IPSV that women aged 40 years and above or living in middle wealth households showed statistically significant reduced adjusted prevalence rates relative to their counterparts aged less than 25 years or living in lower wealth households.

Many of these findings challenge existing studies, raising the need for multifaceted interventions that respond to the unique complexities of IPV and its subtypes in the region’s urban contexts. For instance, research suggests that men with higher educational attainment have more equitable attitudes and practices towards women and may be less violent to their marital partners [57]. Our study suggested otherwise, at least for IPPV. These inconsistencies also imply that there may be other variables operating at the partner- and household-level characteristics that need further exploration. There is, for example, ample evidence that substance and alcohol abuse by male partners can exacerbate violence in the household [58–62].

Among the critical findings in this study is the link between IPV, gender dynamics, and marital power inequities. IPV is often used to maintain and assert power by men [39,63,64]. Situations that diminish men’s sense of control over their female partners can exacerbate their use of violence to affirm control and power [65,66]. Threats to men’s feelings of power in intimate relationships can emerge from diverse sources, including the stresses and disruptions associated with conflicts and post-conflict situations [40,55]. There is evidence that some men may find economically independent and educated female partners threatening. IPV can be part of such threatened men’s strategies to enforce control over female partners [67]. Further, while education can facilitate woman’s recognition and reporting of IPV generally, it can also increase women’s risk for IPV. Educated women may threaten men’s sense of themselves as household heads, decision-makers and breadwinners, especially if such men subscribe to traditional masculine norms regarding the role and authority of men in the household [68]. Also, while previous research shows that economically dependent women are particularly at risk of IPV [69,70], there is also evidence that such women may resort to relationship practices that shield them from violence. Such strategies may include non-confrontation, subservience, and other conflict avoidance strategies [71].

The implications of marital power inequities for IPV in urban SSA are also evident in the positive associations between polygyny and all IPV forms. Polygyny is related to and can boost gender inequities that could promote IPV. Violence toward women can be potentiated by polygyny which often goes together and, in combination with women’s low economic power, low educational attainment, and weak decision-making power. Polygyny enhances male control over women in ways that allow and encourage violence and suppression of rights and liberties of women and girls [72,73]. Additionally, urban women who approved of wife-beating had a significantly elevated prevalence of all forms of IPV relative to their counterparts who did not. Women who approve of wife-beating in unions tend to be poorer, adolescents, or marry early. They are also often powerless and marginalized in their unions, showing inadequate capacity to safely challenge their partners and prevent their abuse or violation. One study in Kenya showed that 36% of girls married before 18 believed that a man is sometimes justified in beating his wife, compared to 20 percent of those who married later [74]. Early ages at cohabitation were associated with increased prevalence rates for IPV. Women who started to cohabit between ages 25 and 35 years had much lower adjusted prevalence rates for all three forms of IPV relative to their counterparts who began to cohabit before age 18. Doku and Asante [75] write that early marriage exacerbates power inequities in unions, putting the woman at extended risks for IPV. Girls who marry before 18 are more likely to experience domestic violence than their peers who marry later. In Peru, where more than half of women report physical or sexual violence, early age at marriage aggravated women’s risk for IPV [76].

The elevated prevalence rates for all IPV forms among women with higher numbers of living children is another critical finding. Large families can be a source of enormous pressure and frustrations that can strain unions and precipitate violence. Research shows that the cost of children is rising in SSA at a time of widespread inflation, unemployment, and growing cost of living [77]. Years of structural adjustment, poor development planning, and decline of public protection and social services have shrunk livelihood opportunities for many urban SSA families, systematically depleting their ability to maintain a decent living and resulting in hardship, poverty and deterioration in the quality of life [13]. These trends have potential to create tensions that can promote IPV [77,78]. Scholars have noted the systematic erosion of the advantages which urban areas have historically enjoyed over rural areas in Africa in terms of livelihood opportunities, social protection, health access to services, and disconnection from traditional gender norms[79–81].

## Conclusion

In just a decade, the majority of SSA women will be urban dwellers. IPV remains a common public health problem and one of the most prevalent forms of violations of women's human rights globally [4,7]. Efforts to address IPV in Africa must include a strong focus on urban areas. The current multi-country study sought to understand the broader dynamics of IPV and its forms in urban SSA using representative data on urban women in the sub-region. The study showed that the prevalence of IPV is highest in conflict and post-conflict states such as DRC and Sierra Leone. It also highlights the complexities and varying influences on IPV subtypes in urban SSA. The factors associated with urban African women’s exposure to different IPV subtypes can be both similar and unique at the same time. Future research should explore the contextual and community-level factors (such as gender norms) that may be associated with IPV against urban women in Africa, as documented in previous research [82]. This study used a cross-sectional dataset and was unable to track trends and changes over time with respect to IPV forms and their correlates in SSA. This limitation notwithstanding, the study reveals some important dynamics, correlates and complexities surrounding IPV in contemporary urban SSA.

## Supporting information

S1 Table Proportions of currently-in-union urban SSA women who have experienced more than a form of IPV. (DOCX)

S2 Table Prevalence estimates of IPPV by age, employment and wealth status of currently-in-union women in urban SSA. (DOCX)

S3 Table Prevalence estimates of IPSV by age, employment and wealth status of currently-in-union women in urban SSA. (DOCX)

S4 Table Prevalence estimates of IPEV by age, employment and wealth status of currently-in-union women in urban SSA. (DOCX)

S5 Table Pearson Chi-square test of IPPV by selected characteristics of urban women in SSA. (DOCX)

S6 Table Pearson Chi-square test of IPSV by selected characteristics of urban women in SSA. (DOCX)

S7 Table Pearson Chi-square test of IPEV by selected characteristics of urban women in SSA. (DOCX)
