# Supplementary material for: Correlates of intimate partner violence among urban women in sub-Saharan Africa
Source: PLoS One. 2020 Mar 25;15(3):e0230508. doi: 10.1371/journal.pone.0230508 (PMC7094863; doi:10.1371/journal.pone.0230508)
Supplement: S1 Table — (DOCX) [file pone.0230508.s001.docx]

Supplementary Tables

Supplementary Table A: Proportions of currently-in-union urban SSA women who have experienced more than a form of IPV

| Country | Year | Total No of Women | Any IPV | Physical and Sexual Violence only | Physical and Emotional  Violence only | Sexual and Emotional Violence only | All IPV |
| --- | --- | --- | --- | --- | --- | --- | --- |
| Angola | 2015-16 | 3,609 | 41.6 | 6.3 | 19.9 | 5.8 | 4.9 |
| Benin | 2017-18 | 1,503 | 38.3 | 4.9 | 13.9 | 5.5 | 4.1 |
| Burkina Faso | 2010 | 1,927 | 18.3 | 1.0 | 6.4 | 1.0 | 0.9 |
| Burundi | 2016-17 | 565 | 32.4 | 9.9 | 12.6 | 7.5 | 6.8 |
| Cameroun | 2011 | 1,576 | 55.9 | 10.2 | 25.7 | 10.2 | 8.2 |
| Chad | 2014-15 | 215 | 30.6 | 9.2 | 14.0 | 8.4 | 7.9 |
| Comoros | 2012 | 652 | 10.8 | 1.0 | 3.2 | 0.8 | 0.4 |
| Congo D. Republic | 2013-14 | 1,439 | 56.3 | 17.5 | 24.0 | 12.8 | 11.1 |
| Cote d’ Ivoire | 2011-12 | 1,704 | 33.8 | 4.8 | 16.3 | 3.8 | 3.5 |
| Ethiopia | 2016 | 632 | 21.2 | 2.4 | 7.7 | 2.3 | 1.6 |
| Gabon | 2012 | 2,555 | 52.8 | 12.8 | 24.0 | 10.4 | 9.6 |
| Gambia | 2013 | 1,472 | 22.1 | 1.2 | 7.5 | 1.1 | 0.9 |
| Kenya | 2014 | 1,296 | 40.7 | 7.9 | 15.5 | 8.0 | 6.0 |
| Malawi | 2015-16 | 694 | 37.2 | 9.3 | 16.1 | 8.1 | 7.3 |
| Mali | 2012-13 | 591 | 42.5 | 8.3 | 18.5 | 8.5 | 7.1 |
| Mozambique | 2011 | 1387 | 50.9 | 6.5 | 22.9 | 5.4 | 4.8 |
| Namibia | 2013 | 491 | 29.3 | 4.2 | 12.6 | 4.5 | 3.3 |
| Nigeria | 2013 | 7,279 | 26.5 | 2.2 | 9.9 | 2.6 | 1.8 |
| Rwanda | 2014-15 | 253 | 28.1 | 6.3 | 11.2 | 7.4 | 5.2 |
| Senegal | 2017 | 841 | 24.0 | 4.5 | 7.4 | 3.1 | 1.5 |
| Sierra Leone | 2013 | 1,073 | 52.7 | 5.7 | 27.3 | 5.5 | 4.9 |
| South Africa | 2016 | 1,101 | 18.8 | 2.2 | 6.1 | 1.9 | 1.6 |
| Tanzania | 2015-16 | 1,836 | 39.7 | 6.9 | 20.1 | 7.1 | 6.0 |
| Togo | 2013-14 | 1,743 | 28.2 | 4.2 | 11.7 | 4.5 | 3.5 |
| Uganda | 2016 | 1,261 | 41.9 | 11.2 | 20.0 | 11.9 | 9.1 |
| Zambia | 2013-14 | 2,871 | 43.5 | 10.6 | 16.7 | 7.6 | 7.0 |
| Zimbabwe | 2015 | 1,577 | 42.7 | 5.7 | 15.7 | 7.9 | 4.8 |
