# Supplementary material for: Correlates of intimate partner violence among urban women in sub-Saharan Africa
Source: PLoS One. 2020 Mar 25;15(3):e0230508. doi: 10.1371/journal.pone.0230508 (PMC7094863; doi:10.1371/journal.pone.0230508)
Supplement: S2 Table — (DOCX) [file pone.0230508.s002.docx]

Supplementary Table B: Prevalence Estimates of IPPV by age, employment and wealth status of currently-in-union women in urban SSA

| Countries |  |  | Age | | | Wealth | | | Employment | |  | |
| --- | --- | --- | --- | --- | --- | --- | --- | --- | --- | --- | --- | --- |
|  | **Year** | **No** | Under-25 years | 25 – 39  years | 40  years + | Lower | Middle | Higher | None | Informal | | Formal |
| Angola | 2015-16 | 3,609 | 37.5 | 31.5 | 28.6 | 33.6 | 35.4 | 26.6 | 34.3 | 31.7 | | 30.8 |
| Benin | 2017-18 | 1,503 | 14.2 | 18.0 | 17.6 | 20.0 | 18.5 | 14.4 | 19.3 | 17.3 | | 6.6 |
| Burkina Faso | 2010 | 1,927 | 9.2 | 14.5 | 10.7 | 11.8 | 13.5 | 12.1 | 9.5 | 14.7 | | 5.1 |
| Burundi | 2016-17 | 565 | 31.6 | 21.9 | 27.7 | 51.6 | 27.7 | 23.2 | 17.0 | 30.3 | | 18.6 |
| Cameroun | 2011 | 1,576 | 42.1 | 43.8 | 37.0 | 43.2 | 41.0 | 43.7 | 37.2 | 44.3 | | 43.2 |
| Chad | 2014-15 | 215 | 19.3 | 26.6 | 25.2 | 34.0 | 38.3 | 20.7 | 20.5 | 30.1 | | 22.1 |
| Comoros | 2012 | 652 | 7.9 | 6.9 | 3.6 | 6.6 | 7.9 | 4.8 | 7.0 | 6.7 | | 5.1 |
| Congo D. Republic | 2013-14 | 1,439 | 48.4 | 44.0 | 44.2 | 44.6 | 45.6 | 44.6 | 39.9 | 48.5 | | 36.9 |
| Cote d’ Ivoire | 2011-12 | 1,704 | 30.0 | 28.2 | 25.9 | 49.8 | 28.6 | 27.3 | 21.7 | 31.8 | | 12.6 |
| Ethiopia | 2016 | 632 | 12.5 | 11.8 | 14.4 | 10.3 | 12.5 | 12.5 | 11.2 | 14.9 | | 8.6 |
| Gabon | 2012 | 2,555 | 47.7 | 42.7 | 42.7 | 55.0 | 38.7 | 40.2 | 43.1 | 45.6 | | 39.4 |
| Gambia | 2013 | 1,472 | 9.4 | 17.9 | 14.0 | 12.0 | 17.6 | 12.2 | 11.8 | 18.5 | | 15.0 |
| Kenya | 2014 | 1,296 | 24.1 | 30.1 | 29.3 | 41.0 | 32.0 | 23.1 | 18.6 | 36.1 | | 18.1 |
| Malawi | 2015-16 | 694 | 27.2 | 27.7 | 12.0 | 25.6 | 30.9 | 23.9 | 25.9 | 25.5 | | 24.4 |
| Mali | 2012-13 | 591 | 29.6 | 27.4 | 26.4 | 30.8 | 26.3 | 28.4 | 23.8 | 33.8 | | 43.3 |
| Mozambique | 2011 | 1387 | 34.9 | 37.4 | 26.2 | 26.8 | 36.7 | 34.6 | 36.6 | 33.9 | | 19.7 |
| Namibia | 2013 | 491 | 30.0 | 18.9 | 17.8 | 31.9 | 22.7 | 13.4 | 28.4 | 18.8 | | 13.6 |
| Nigeria | 2013 | 7,279 | 11.8 | 16.0 | 16.1 | 11.6 | 15.5 | 15.8 | 8.7 | 17.8 | | 13.3 |
| Rwanda | 2014-15 | 253 | 12.4 | 19.7 | 18.7 | 40.5 | 34.0 | 13.4 | 7.1 | 22.5 | | 8.7 |
| Senegal | 2017 | 841 | 17.0 | 18.2 | 8.2 | 18.0 | 17.4 | 11.6 | 13.5 | 17.5 | | 6.8 |
| Sierra Leone | 2013 | 1,073 | 42.4 | 47.9 | 38.2 | 28.0 | 44.3 | 45.9 | 38.5 | 47.9 | | 36.2 |
| South Africa | 2016 | 1,101 | 23.2 | 10.2 | 8.5 | 16.3 | 14.0 | 3.4 | 12.8 | 12.5 | | 6.2 |
| Tanzania | 2015-16 | 1,836 | 27.2 | 30.0 | 35.2 | 35.1 | 36.6 | 26.9 | 28.6 | 32.6 | | 17.2 |
| Togo | 2013-14 | 1,743 | 17.1 | 17.0 | 13.6 | 46.6 | 20.1 | 12.4 | 15.3 | 16.7 | | 4.2 |
| Uganda | 2016 | 1,261 | 20.6 | 29.3 | 36.4 | 48.1 | 38.2 | 19.9 | 24.6 | 31.9 | | 18.1 |
| Zambia | 2013-14 | 2,871 | 38.4 | 37.3 | 33.4 | 43.0 | 40.4 | 32.7 | 34.5 | 40.2 | | 33.2 |
| Zimbabwe | 2015 | 1,577 | 29.9 | 27.6 | 24.9 | ---- | 34.6 | 22.2 | 25.4 | 32.0 | | 18.4 |

*Note: None reported to be in lower wealth category in Zimbabwe*
