# Supplementary material for: Correlates of intimate partner violence among urban women in sub-Saharan Africa
Source: PLoS One. 2020 Mar 25;15(3):e0230508. doi: 10.1371/journal.pone.0230508 (PMC7094863; doi:10.1371/journal.pone.0230508)
Supplement: S3 Table — (DOCX) [file pone.0230508.s003.docx]

Supplementary Table C: Prevalence Estimates of IPSV by age, employment and wealth status of currently-in-union women in urban SSA

| Countries |  |  | Age | | | Wealth | | | Employment | |  |
| --- | --- | --- | --- | --- | --- | --- | --- | --- | --- | --- | --- |
|  | **Year** | **No** | Under-25 years | 25 – 39  years | 40 years+ | Lower | Middle | Higher | None | Informal | Formal |
| Angola | 2015-16 | 3,609 | 11.6 | 7.5 | 4.2 | 11.7 | 8.3 | 5.8 | 8.9 | 7.5 | 6.9 |
| Benin | 2017-18 | 1,503 | 4.7 | 9.3 | 5.1 | 9.3 | 9.5 | 4.9 | 6.8 | 7.9 | 4.1 |
| Burkina Faso | 2010 | 1,927 | 1.0 | 2.1 | 1.6 | 1.1 | 1.8 | 1.7 | 1.5 | 1.8 | 1.6 |
| Burundi | 2016-17 | 565 | 18.8 | 11.4 | 13.8 | 28.9 | 24.3 | 11.1 | 6.3 | 19.0 | 5.3 |
| Cameroun | 2011 | 1,576 | 17.6 | 13.7 | 8.8 | 20.4 | 14.4 | 13.0 | 11.4 | 15.1 | 14.4 |
| Chad | 2014-15 | 215 | 14.8 | 11.5 | 3.6 | 10.2 | 18.5 | 10.4 | 12.5 | 10.3 | 0.0 |
| Comoros | 2012 | 652 | 1.8 | 2.2 | 0.3 | 1.0 | 1.5 | 2.5 | 2.3 | 1.9 | 0.0 |
| Congo D. Republic | 2013-14 | 1,439 | 19.6 | 22.5 | 22.2 | 26.4 | 26.5 | 18.1 | 15.8 | 25.3 | 19.7 |
| Cote d’ Ivoire | 2011-12 | 1,704 | 6.6 | 5.7 | 3.7 | 49.8 | 5.7 | 4.8 | 4.7 | 6.0 | 2.2 |
| Ethiopia | 2016 | 632 | 2.8 | 4.2 | 4.2 | 12.9 | 12.4 | 3.1 | 2.8 | 6.2 | 0.3 |
| Gabon | 2012 | 2,555 | 16.5 | 12.9 | 17.3 | 21.2 | 11.1 | 13.3 | 12.9 | 16.9 | 11.9 |
| Gambia | 2013 | 1,472 | 2.0 | 1.6 | 1.4 | 0.0 | 2.9 | 0.4 | 0.7 | 2.4 | 4.6 |
| Kenya | 2014 | 1,296 | 7.2 | 12.8 | 10.4 | 15.4 | 11.6 | 9.8 | 4.7 | 15.0 | 8.1 |
| Malawi | 2015-16 | 694 | 20.9 | 12.8 | 3.4 | 35.4 | 20.7 | 10.1 | 9.4 | 19.5 | 8.0 |
| Mali | 2012-13 | 591 | 10.3 | 11.8 | 8.6 | 30.8 | 11.0 | 10.8 | 10.3 | 12.3 | 0.0 |
| Mozambique | 2011 | 1,387 | 9.6 | 8.3 | 4.8 | 4.5 | 9.3 | 7.8 | 8.5 | 7.7 | 6.9 |
| Namibia | 2013 | 491 | 12,7 | 6.1 | 3.2 | 10.2 | 9.0 | 2.2 | 10.7 | 6.0 | 2.4 |
| Nigeria | 2013 | 7,279 | 3.9 | 3.7 | 3.1 | 5.5 | 4.0 | 2.9 | 3.6 | 3.9 | 1.8 |
| Rwanda | 2014-15 | 253 | 7.7 | 14.4 | 11.7 | 15.8 | 15.7 | 12.1 | 21.7 | 11.7 | 0.0 |
| Senegal | 2017 | 841 | 12.3 | 8.2 | 7.5 | 10.0 | 9.0 | 8.0 | 6.1 | 10.8 | 4.7 |
| Sierra Leone | 2013 | 1,073 | 10.2 | 7.8 | 4.0 | 6.2 | 6.2 | 7.8 | 6.0 | 7.7 | 7.8 |
| South Africa | 2016 | 1,101 | 9.3 | 2.7 | 1.3 | 4.1 | 3.3 | 1.8 | 3.4 | 3.0 | 2.2 |
| Tanzania | 2015-16 | 1,836 | 10.3 | 9.5 | 7.7 | 13.8 | 11.0 | 7.9 | 8.2 | 10.0 | 6.4 |
| Togo | 2013-14 | 1,743 | 4.8 | 6.6 | 2.6 | 2.6 | 7.2 | 4.1 | 5.1 | 5.7 | 0.0 |
| Uganda | 2016 | 1,261 | 13.8 | 17.6 | 16.2 | 22.2 | 20.9 | 13.4 | 17.6 | 17.3 | 11.2 |
| Zambia | 2013-14 | 2,871 | 12.9 | 12.8 | 14.1 | 20.3 | 13.7 | 11.8 | 10.6 | 16.0 | 10.4 |
| Zimbabwe | 2015 | 1,577 | 14.2 | 10.7 | 5.5 | ---- | 14.0 | 7.8 | 9.2 | 12.4 | 7.0 |

*Note: None reported to be in lower wealth category in Zimbabwe*
