# Supplementary material for: Correlates of intimate partner violence among urban women in sub-Saharan Africa
Source: PLoS One. 2020 Mar 25;15(3):e0230508. doi: 10.1371/journal.pone.0230508 (PMC7094863; doi:10.1371/journal.pone.0230508)
Supplement: S4 Table — (DOCX) [file pone.0230508.s004.docx]

Supplementary Table D: Prevalence Estimates of IPEV by age, employment and wealth status of currently-in-union women in urban SSA

| Countries |  |  | Age | | | Wealth | | | Employment | |  |
| --- | --- | --- | --- | --- | --- | --- | --- | --- | --- | --- | --- |
|  | Year | **No** | Under-25 years | 25-39  years | 40 years+ | Lower | Middle | Higher | None | Informal | Formal |
| Angola | 2015-16 | 3,609 | 29.5 | 27.9 | 27.4 | 27.7 | 28.6 | 27.7 | 28.6 | 26.8 | 36.2 |
| Benin | 2017-18 | 1,503 | 29.5 | 35.0 | 34.6 | 36.9 | 35.9 | 30.4 | 34.6 | 34.5 | 23.0 |
| Burkina Faso | 2010 | 1,927 | 9.9 | 13.4 | 9.9 | 12.2 | 10.8 | 12.1 | 10.6 | 12.6 | 10.6 |
| Burundi | 2016-17 | 565 | 22.9 | 17.0 | 17.5 | 26.9 | 31.5 | 16.0 | 15.2 | 23.9 | 5.4 |
| Cameroun | 2011 | 1,576 | 35.7 | 38.7 | 37.8 | 37.6 | 37.4 | 38.0 | 29.2 | 41.0 | 45.5 |
| Chad | 2014-15 | 215 | 19.0 | 20.7 | 12.5 | 27.4 | 30.7 | 16.0 | 16.1 | 23.8 | 12.8 |
| Comoros | 2012 | 652 | 6.3 | 7.6 | 6.4 | 6.7 | 9.5 | 3.9 | 6.1 | 10.8 | 6.5 |
| Congo D. Republic | 2013-14 | 1,439 | 34.7 | 32.1 | 32.4 | 34.5 | 36.2 | 30.2 | 34.5 | 32.5 | 25.0 |
| Cote d’ Ivoire | 2011-12 | 1,704 | 20.2 | 22.0 | 21.4 | 49.8 | 22.7 | 19.6 | 14.7 | 24.7 | 13.9 |
| Ethiopia | 2016 | 632 | 16.3 | 13.7 | 22.0 | 22.9 | 16.4 | 15.3 | 13.8 | 19.6 | 9.3 |
| Gabon | 2012 | 2,555 | 29.9 | 32.8 | 32.6 | 35.5 | 28.9 | 35.0 | 30.9 | 33.3 | 32.7 |
| Gambia | 2013 | 1,472 | 10.2 | 15.4 | 16.3 | 13.7 | 13.0 | 16.0 | 13.7 | 15.4 | 10.2 |
| Kenya | 2014 | 1,296 | 20.3 | 27.9 | 29.1 | 36.8 | 30.6 | 20.4 | 17.1 | 32.5 | 19.4 |
| Malawi | 2015-16 | 694 | 24.9 | 25.4 | 20.6 | 29.6 | 32.3 | 22.2 | 28.8 | 28.7 | 10.8 |
| Mali | 2012-13 | 591 | 33.5 | 29.6 | 37.8 | -- | 28.7 | 32.9 | 30.2 | 34.5 | 39.9 |
| Mozambique | 2011 | 1387 | 35.3 | 42.9 | 30.9 | 40.0 | 39.2 | 37.3 | 34.2 | 45.1 | 26.9 |
| Namibia | 2013 | 491 | 27.0 | 19.8 | 20.1 | 20.6 | 22.7 | 19.0 | 23.5 | 20.8 | 18.3 |
| Nigeria | 2013 | 7,279 | 15.9 | 21.7 | 20.6 | 21.4 | 20.3 | 20.5 | 14.5 | 22.1 | 22.7 |
| Rwanda | 2014-15 | 253 | 19.2 | 17.6 | 10.4 | 28.7 | 31.2 | 12.6 | 14.0 | 18.3 | 5.2 |
| Senegal | 2017 | 841 | 11.9 | 14.6 | 12.4 | 11.2 | 14.7 | 12.4 | 12.5 | 14.6 | 10.8 |
| Sierra Leone | 2013 | 1,073 | 29.5 | 37.2 | 31.2 | 21.9 | 32.8 | 36.3 | 32.0 | 36.9 | 15.9 |
| South Africa | 2016 | 1,101 | 21.9 | 12.4 | 12.4 | 16.9 | 12.5 | 11.6 | 15.7 | 13.7 | 8.1 |
| Tanzania | 2015-16 | 1,836 | 24.4 | 27.8 | 32.8 | 35.6 | 32.9 | 24.9 | 23.3 | 30.0 | 26.8 |
| Togo | 2013-14 | 1,743 | 21.8 | 23.9 | 22.6 | 54.2 | 28.3 | 18.4 | 18.0 | 24.7 | 9.7 |
| Uganda | 2016 | 1,261 | 28.0 | 32.8 | 36.3 | 45.8 | 44.2 | 24.4 | 24.6 | 38.4 | 19.8 |
| Zambia | 2013-14 | 2,871 | 19.3 | 22.1 | 21.1 | 32.2 | 24.3 | 17.0 | 20.0 | 22.4 | 22.5 |
| Zimbabwe | 2015 | 1,577 | 30.5 | 28.2 | 31.5 | -- | 33.9 | 25.7 | 26.8 | 32.4 | 24.7 |

*None reported to be in lower wealth category in Zimbabwe*
