# Supplementary material for: Correlates of intimate partner violence among urban women in sub-Saharan Africa
Source: PLoS One. 2020 Mar 25;15(3):e0230508. doi: 10.1371/journal.pone.0230508 (PMC7094863; doi:10.1371/journal.pone.0230508)
Supplement: S5 Table — (DOCX) [file pone.0230508.s005.docx]

Supplementary Table E: Pearson Chi-square test of IPPV by selected characteristics of urban women in SSA

| Country | Individual Variables | | | | | | Spouse / Partner’s Variables | | | Household Variables | |
| --- | --- | --- | --- | --- | --- | --- | --- | --- | --- | --- | --- |
|  | **Age** | **Education** | **Occupation** | **Age at First**  **Cohabitation** | **No. of Living Children** | **Wife beating**  **justified** | **Age** | **Education** | **Occupation** | **Household Wealth** | **Number of Wives** |
| Angola | 12.45* | 1.46 | 2.01 | 22.37* | 3.98 | 0.21 | 8.77 | 3.46 | 0.09 | 19.84* | 0.01 |
| Benin | 2.70 | 24.47* | 8.32 | 23.73* | 32.09* | 31.32* | 3.50 | 25.61* | 3.85 | 6.96 | 13.81* |
| Burkina Faso | 13.19 | 14.20* | 23.99* | 20.18* | 25.87* | 10.17 | 7.81 | 20.75* | 7.11 | 0.93 | 1.00 |
| Burundi | 8.08 | 23.37* | 22.26* | 37.63* | 11.54 | 23.55* | 17.11* | 15.90 | 16.64 * | 13.95* | 3.54 |
| Cameroun | 4.04 | 11.86* | 6.97 | 30.07* | 6.92 | 14.09* | 15.61* | 16.18* | 3.84 | 1.16 | 4.18 |
| Chad | 1.73 | 4.37 | 3.42 | 4.01 | 2.17 | 0.19 | 4.32 | 2.52 | 0.09 | 6.76 | 2.28 |
| Comoros | 3.21 | 14.32* | 0.88 | 3.86 | 16.22* | 5.58 | 6.39 | 1.67 | 0.18 | 2.93 | 2.52 |
| Congo D. Republic | 1.82 | 7.44 | 11.44 | 32.25* | 2.25 | 24.95* | 13.89* | 0.55 | 48.99* | 0.14 | 10.27* |
| Cote d’ Ivoire | 1.43 | 6.79 | 26.83* | 18.37* | 4.26 | 8.86* | 2.61 | 3.57 | 8.05 | 1.88 | 5.42 |
| Ethiopia | 0.85 | 10.84 | 5.21 | 6.57 | 5.29 | 0.63 | 5.82 | 29.93* | 23.39* | 0.21 | 23.54* |
| Gabon | 3.53 | 28.40* | 3.75 | 49.85* | 13.62 | 56.19* | 28.72* | 5.85 | 7.56 | 46.02* | 8.18* |
| Gambia | 14.32* | 9.46 | 11.47 | 3.26 | 22.37* | 26.92* | 7.10 | 19.69 | 8.75 | 8.12 | 3.29 |
| Kenya | 4,22 | 19.23* | 52.13* | 6.77 | 56.44* | 16.65* | 7.35 | 21.36* | 15.78* | 25.92* | 15.18* |
| Malawi | 12.98 | 33.87* | 0.14 | 3.27 | 2.65 | 0.00 | 7.47 | 9.91 | 0.98 | 3.45 | 0.02 |
| Mali | 0.54 | 0.36 | 11.05* | 0.97 | 3.90 | 1.23 | 0.52 | 2.01 | --- | 0.36 | 0.65 |
| Mozambique | 11.75* | 5.53 | 10.12* | 14.79* | 12.30 | 2.28 | 5.97 | 3.54 | 3.45 | 5.72 | 1.18 |
| Namibia | 6.10 | 7.09 | 14.34* | 9.05 | 14.53* | 4.66 | 3.62 | 2.10 | 6.43 | 17.20* | 1.39 |
| Nigeria | 13.94* | 156.67* | 83.09* | 39.72* | 23.67* | 50.04* | 0.70 | 123.17* | 20.73* | 7.81 | 0.90 |
| Rwanda | 1.51 | 14.29* | 10.04* | 22.01* | 11.19* | 4.85 | 1.36 | 19.69* | 8.28 | 20.30* | 0.01 |
| Senegal | 14.01 | 2.48 | 6.65 | 7.58 | 6.35 | 4.99 | 4.70 | 7.56 | 9.55 | 5.94 | 0.36 |
| Sierra Leone | 8.93 | 7.80 | 10.75 | 8.98 | 25.54* | 3.91 | 3.34 | 3.85 | 5.77 | 6.69 | 4.89 |
| South Africa | 17.43* | 7.08 | 8.08 | 0.38 | 5.55 | 57.60* | 16.51* | 1.15 | 21.04* | 30.70* | 0.71 |
| Tanzania | 5.47 | 3.39 | 13.07* | 15.16* | 15.97 | 11.38* | 1.01 | 20.27* | 0.62 | 16.60* | 6.24 |
| Togo | 2.24 | 0.71 | 3.65 | 22.44* | 4.65 | 4.23 | 5.25 | 4.78 | 7.68 | 32.72* | 1.35 |
| Uganda | 16.50* | 52.29* | 18.26* | 23.89* | 48.01* | 23.34* | 17.00* | 47.83* | 31.63* | 72.31* | 17.57* |
| Zambia | 3.42 | 38.56* | 11.70* | 16.60* | 23.22* | 84.24* | 7.67 | 8.66 | 28.23* | 21.20* | 0.89 |
| Zimbabwe | 2.27 | 10.00* | 24.30* | 47.97* | 15.57* | 16.38* | 4.07 | 20.59* | 9.45 | 37.69* | 12.16* |

*Significance level: p<0.05; Only one job category reported for husbands/partners in Mali*
