# Supplementary material for: Correlates of intimate partner violence among urban women in sub-Saharan Africa
Source: PLoS One. 2020 Mar 25;15(3):e0230508. doi: 10.1371/journal.pone.0230508 (PMC7094863; doi:10.1371/journal.pone.0230508)
Supplement: S6 Table — (DOCX) [file pone.0230508.s006.docx]

Supplementary Table F: Pearson Chi-square test of IPSV by selected characteristics of urban women in SSA

| Country | Individual Variables | | | | | Spouse / Partner’s Variables | | Household Variables | | | |
| --- | --- | --- | --- | --- | --- | --- | --- | --- | --- | --- | --- |
|  | **Age** | **Education** | **Occupation** | **Age at First**  **Cohabitation** | **No. of Living Children** | **Wife beating**  **justified** | **Age** | **Education** | **Occupation** | **Household Wealth** | **Number of Wives** |
| Angola | 23.97* | 4.30 | 1.76 | 2.73 | 4.06 | 10.01 | 12.52* | 5.65 | 11.38 | 11.73 | 1.55 |
| Benin | 11.95* | 12.01* | 2.10 | 14.10* | 15.45* | 9.99* | 5.49 | 12.47 | 0.58 | 12.82* | 3.01 |
| Burkina Faso | 2.97 | 10.30 | 0.34 | 0.79 | 8.43 | 6.55 | 1.28 | 4.35 | 2.67 | 0.29 | 0.06 |
| Burundi | 6.43 | 35.86* | 39.15* | 23.91* | 9.19* | 27.19* | 17.43* | 15.54* | 2.92 | 22.81* | 5.28 |
| Cameroun | 11.08* | 13.97* | 3.70 | 11.27* | 4.06 | 25.30* | 9.64* | 6.32 | 0.56 | 2.09 | 0.01 |
| Chad | 3.85 | 1.19 | 1.53 | 1.07 | 7.95 | 2.76 | 1.27 | 6.58 | 6.60 | 1.99 | 0.68 |
| Comoros | 2.46 | 14.77* | 4.31 | 3.71 | 2.48 | 1.00 | 1.36 | 0.01 | 2.69 | 1.88 | 3.52 |
| Congo D. Republic | 1.12 | 14.65* | 16.17* | 6.71 | 1.56 | 10.49* | 0.47 | 2.29 | 50.99* | 14.39* | 7.54* |
| Cote d’ Ivoire | 3.06 | 21.36* | 2.86 | 12.72 | 5.56 | 22.07* | 3.76 | 7.98 | 2.27 | 25.19* | 0.88 |
| Ethiopia | 0.77 | 3.27 | 13.15* | 18.64* | 5.45 | 1.91 | 0.11 | 18.97 | 13.42* | 18.58* | 60.31* |
| Gabon | 7.81* | 10.01* | 9.25* | 41.52* | 3.49 | 21.63* | 22.49* | 1.63 | 6.70* | 39.65* | 1.40 |
| Gambia | 0.47 | 4.88 | 11.10* | 0.90 | 10.25* | 5.14* | 5.81 | 9.62* | 2.26 | 16.08* | 1.18 |
| Kenya | 7.80 | 4.54 | 29.97* | 2.88 | 4.68 | 11.57* | 5.01 | 6.19 | 3.56 | 4.34 | 22.30* |
| Malawi | 20.06* | 21.25* | 20.36* | 13.47* | 3.11 | 0.68 | 7.43 | 21.79* | 1.85 | 29.93* | 1.46 |
| Mali | 1.22 | 0.81 | 2.31 | 4.38 | 3.88 | 1.45 | 0.74 | 4.25 | --- | 0.95 | 2.66 |
| Mozambique | 5.78 | 6.03 | 0.55 | 9.25 | 2.12 | 17.92* | 3.82 | 6.07 | 12.50* | 4.25 | 0.09 |
| Namibia | 9.0 | 11.95* | 12.12* | 12.96* | 1.03 | 4.82 | 5.89 | 6.19 | 19.60* | 14.18* | 5.35 |
| Nigeria | 1.70 | 1.03 | 8.57 | 19.96* | 5.82 | 96.65* | 5.54 | 5.17 | 1.70 | 13.63 | 1.77 |
| Rwanda | 1.72 | 8.52* | 8.63 | 7.81* | 0.99 | 11.03 | 1.36 | 3.25 | 2.17 | 0.69 | 0.27 |
| Senegal | 3.11 | 7.61 | 7.06 | 0.78 | 12.20 | 0.23 | 2.19 | 0.48 | 9.00 | 0.42 | 1.12 |
| Sierra Leone | 8.06 | 10.75 | 1.16 | 16.18 | 1.64 | 18.96* | 1.55 | 1.70 | 6.72 | 1.14 | 2.75 |
| South Africa | 16.60* | 0.85 | 0.91 | 8.62 | 2.00 | 79.26* | 14.16 | 1.74 | 8.79 | 2.99 | 0.51 |
| Tanzania | 1.53 | 2.99 | 2.37 | 6.07 | 6.79 | 3.82 | 0.60 | 13.60* | 6.98* | 6.57 | 12.95* |
| Togo | 8.28* | 3.05 | 1.96 | 10.54 | 2.92 | 3.18* | 10.10* | 7.47* | 0.01 | 8.01* | 0.09 |
| Uganda | 2.61 | 22.25* | 5.10 | 1.47 | 22.56* | 27.73* | 0.46 | 21.59* | 4.23 | 13.63* | 18.90* |
| Zambia | 0.62 | 10.57* | 20.72* | 13.83 | 10.14 | 60.85* | 4.05 | 9.53 | 1.74 | 6.93 | 2.51 |
| Zimbabwe | 14.79* | 1.35 | 8.99 | 28.68* | 3.60 | 6.62 | 9.16 | 8.53 | 17.19* | 20.12* | 32.89* |

*Significance level: p<0.05; Only one job category reported for husbands/partners in Mali*
