# Supplementary material for: Correlates of intimate partner violence among urban women in sub-Saharan Africa
Source: PLoS One. 2020 Mar 25;15(3):e0230508. doi: 10.1371/journal.pone.0230508 (PMC7094863; doi:10.1371/journal.pone.0230508)
Supplement: S7 Table — (DOCX) [file pone.0230508.s007.docx]

Supplementary Table G: Pearson Chi-square test of IPEV by selected characteristics of urban women in SSA

| Country | Individual Variables | | | | | | Spouse / Partner’s Variables | | | Household Variables | |
| --- | --- | --- | --- | --- | --- | --- | --- | --- | --- | --- | --- |
|  | **Age** | **Education** | **Occupation** | **Age at First**  **Cohabitation** | **No. of Living Children** | **Wife beating**  **Justified** | **Age** | **Education** | **Occupation** | **Household Wealth** | **Number of Wives** |
| Angola | 0.88 | 3.25 | 9.34 | 4.16 | 1.21 | 0.59 | 1.29 | 14.43* | 4.34 | 0.25 | 0.41 |
| Benin | 3.61 | 8.21 | 5.03 | 14.92 | 23.83* | 0.58 | 8.13 | 14.93* | 11.05* | 6.72 | 16.10* |
| Burkina Faso | 7.22 | 23.29* | 2.24 | 3.36 | 26.02* | 36.22* | 6.67 | 1.75 | 12.78 | 0.81 | 0.03 |
| Burundi | 3.14 | 54.53* | 36.26* | 45.45* | 15.24* | 64.03* | 23.09* | 21.90* | 26.72* | 18.11* | 6.90* |
| Cameroun | 1.15 | 11.20* | 21.52* | 3.69 | 11.72 | 2.18 | 1.33 | 22.79* | 4.51 | 0.06 | 0.16 |
| Chad | 1.55 | 0.28 | 2.88 | 3.59 | 3.42 | 1.41 | 2.32 | 1.06 | 0.30 | 5.78 | 0.02 |
| Comoros | 0.53 | 3.75 | 4.45 | 6.48 | 17.71* | 1.10 | 6.81 | 1.90 | 2.74 | 8.48* | 0.17 |
| Congo D. Republic | 0.70 | 4.71 | 3.14 | 11.51 | 16.40 | 17.74* | 2.69 | 2.66 | 12.42* | 5.28 | 4.87* |
| Cote d’ Ivoire | 0.49 | 5.45 | 21.55* | 9.22 | 2.71 | 11.46* | 3.49 | 6.18 | 0.49 | 5.37 | 2.35 |
| Ethiopia | 7.14 | 29.55* | 11.14 | 1.05 | 7.04 | 0.02 | 8.14 | 25.45* | 16.25 | 1.91 | 1.04 |
| Gabon | 1.47 | 10.51* | 1.48 | 55.19* | 35.21* | 18.83* | 7.86* | 2.38 | 7.64* | 11.69* | 18.25* |
| Gambia | 6.97* | 4.29 | 1.82 | 3.22 | 25.19* | 0.45 | 9.55* | 0.36 | 5.55 | 2.75 | 0.59 |
| Kenya | 8.13 | 4.24 | 37.46* | 3.56 | 16.27 | 22.31* | 4.77* | 6.51 | 7.18 | 26.87* | 11.11* |
| Malawi | 1.22 | 33.50* | 25.98* | 10.41 | 5.67 | 10.15 | 0.53 | 11.46* | 4.54 | 7.91 | 0.35 |
| Mali | 3.67 | 3.55 | 1.95 | 1.95 | 1.24 | 0.55 | 1.31 | 5.05 | --- | 6.25 | 1.25 |
| Mozambique | 15.84* | 12.61* | 23.84* | 4.56 | 20.27* | 0.73 | 14.85* | 7.12 | 1.12 | 0.80 | 4.16 |
| Namibia | 2.49 | 9.53 | 1.66 | 4.24 | 7.17 | 11.56 | 2.40 | 2.96 | 2.95 | 1.13 | 3.14 |
| Nigeria | 20.11* | 79.52* | 47.45* | 16.99* | 35.88* | 77.01* | 2.17 | 74.95* | 2.80 | 0.46 | 0.45 |
| Rwanda | 2.15 | 4.67 | 3.15 | 1.71 | 1.45 | 0.06 | 0.28 | 10.13 | 5.73 | 12.99* | 6.66* |
| Senegal | 1.13 | 3.33 | 1.26 | 4.90 | 10.54 | 5.68 | 0.35 | 8.18 | 11.09* | 1.29 | 0.26 |
| Sierra Leone | 6.14 | 5.01 | 11.83 | 3.46 | 32.29* | 20.29* | 3.98 | 7.16 | 19.77* | 5.55 | 2.67 |
| South Africa | 7.14 | 1.33 | 8.26 | 4.97 | 0.60 | 61.38* | 3.10 | 0.14 | 5.26 | 4.00 | 0.04 |
| Tanzania | 6.07 | 2.01 | 6.92 | 3.52 | 23.87* | 5.34 | 1.17 | 7.89 | 0.80 | 13.83* | 10.68* |
| Togo | 0.65 | 9.11 | 9.11* | 11.28* | 12.99* | 15.31* | 2.16 | 6.65 | 3.70 | 33.94* | 1.42 |
| Uganda | 4.28 | 40.83* | 36.67* | 5.88 | 32.33* | 11.82* | 9.52* | 45.86* | 29.74* | 55.35* | 6.89* |
| Zambia | 2.57 | 5.55 | 2.74 | 8.01 | 29.32* | 15.81* | 3.31 | 17.53* | 16.67* | 31.66* | 0.15 |
| Zimbabwe | 1.81 | 11.52 | 9.69* | 19.34* | 3.04 | 14.59* | 1.97 | 8.81 | 8.39 | 15.76* | 26.16* |

*Significance level: p<0.05; Only one job category reported for husbands/partners in Mali*
